# Distinctive phenogroup to differentiate diagnosis of cardiac myxoma vs cardiovascular disease examining blood-based circulating cell biomarkers

**DOI:** 10.1038/s41598-023-47639-y

**Published:** 2023-11-21

**Authors:** Giuseppe Donato, Chiara Mignogna, Gianluca Santise, Ivan Presta, Teresa Ferrazzo, Virginia Garo, Daniele Maselli, Antonio Curcio, Salvatore De Rosa, Carmen Spaccarotella, Vincenzo Mollace, Francesco Gentile, Ciro Indolfi, Natalia Malara

**Affiliations:** 1 Department of Health Sciences, University “Magna Graecia”, 88100 Catanzaro, Italy; 2 Interdipartimentale Service Center, University Magna Graecia, Catanzaro, Italy; 3 Cardiothoracic Surgery Unit, Sant’Anna Hospital, Via Pio X, 111, Catanzaro, Italy; 4 Department of Medical and Surgical Sciences, University Magna Graecia, Catanzaro, Italy; 5 Department of Experimental and Clinical Medicine, University Magna Graecia, Catanzaro, Italy

**Keywords:** Biomarkers, Oncology

## Abstract

Cardiac myxoma (CM) is a potentially life-threatening disease because frequently asymptomatic or debuts with aspecific manifestations. Definitive diagnosis is established by histopathological assessment including tumor and endothelial cell markers. To derive a specific panel of circulating cells antigenically detectable, pre-surgery peripheral blood samples of CM patients were analyzed. Pre-surgery peripheral blood samples from patients with CM were simultaneously analyzed for Circulating tumor cells (CTCs) and circulating endothelial cells (CECs) that were matched with tumor tissue profiles and with patient-derived xenografts (PDXs) distinguishing tumor regions. Moreover, CECs values in CM patients were further matched with CEC’s levels in cardiovascular disease and control subjects. The blood-derived cytological specimens detected at least 1–3 CTCs/ml in 10 tested CM samples (p = 0.0001) showing specific CM features preserved in the central zones of the tumor. The central zone of the primary tumor, supported by a vessel density rate (55 ± 7%), with a proliferative profile of 32 ± 3% and a percentage of Calretinin^pos^ cells (p = 0.03), is the principal site of CTCs (r = 00) dissemination. The subsets of endothelial cells recognized in the blood were indifferent to their topological distribution within the tumor and corresponding PDXs. With further refinement and validation in large cohorts, multiparametric liquid biopsies can optimally integrate clinically informative datasets and maximize their utility in pre-surgery evaluation of CM patients. Blood-derived culture’s protocol provides a versatile method capable of viable analysis of CTCs of non-hematological rare tumors which conventional antibody-mediated analytical platform is unable to perform. Distinctive blood- based cell phenotype contributes to differentiate CM from other differentials assuring its prompt surgical resection by combining blood-based cell biomarkers integrated with clinically informative datasets.

## Introduction

Cardiac myxoma (CM) is the most frequent primary cardiac tumor, representing the 83% of all the neoplasms of this type. The incidence of CM in autopsies has been estimated at 0.03%^1^. They may be isolated or sporadic, or instead familial or part of a syndrome, such as the Carney complex. In CMs, mutations inactivating the tumor suppressor gene PRKAR1A—both in sporadic and familiar forms—are very common. The PRKAR1A gene encodes the regulatory subunit type 1α (R1α) of the protein kinase A (PKA)^2,3^.

The majority of CMs grow in the left atrium, mainly in the region of the fossa ovalis. In fact, for a long time, such tumors were thought to arise from microscopic endocardial/endothelial structures known as Prichard structures, i.e. little endocardial deformities with capillary spaces lined with plump endothelial cells, located in the interatrial septum^1^.

Moreover, CMs are mainly detected in females with a mean age at the diagnosis of about 50 years. When CMs are part of the Carney complex (CC) syndrome, they can occur among people younger than 50, and emerge in multiple sites of the heart^3^.

Grossly, the appearance of CMs is that of smooth, round, and compact masses or, alternatively, of friable and villous vegetation. The location and general characteristics of CMs are important factors for many clinical aspects of the disease: in fact, such neoplasms may cause strokes, peripheral embolisms, syncope, or sudden death. Conversely, true metastases are considered extremely rare^2,4^.

Constitutional symptoms like fever, high erythrocyte sedimentation rate and anemia can be present. Furthermore, CMs can occasionally produce interleukins 6 (an interleukin that acts as both a pro-inflammatory cytokine and an anti-inflammatory myokine) and dense and heterogeneous systems of adaptive and innate immunity cells^5,6^.

Microscopically, CM is a neoplasm composed of plump and stellate cells, and of morphologically bland mesenchymal cells set in the myxoid stroma.

Myxoma cells, in turn, may form rings, cords and nests that are often closely associated with capillaries. Interestingly, vascular channels often appear to develop from myxomatous structures^2^.

Sub-endothelial reservoir cells or cardiomyocyte progenitors have been formerly suggested as possible precursors of CMs cells.

Another possible origin of CMs is the Cardiac Neural Crests (CNCs), suggested from the expression of calretinin in the tumor cells. The hypothesis of CNCs as origin of CMs is also supported by the expression of other markers such as Ubiquitin carboxyl-terminal hydrolase isozyme L1 (PGP9.5), S100 proteins and neuron-specific enolase (NSE), semaphorin3C and plexinA2^3,7,8^.

Since, at the best of our knowledge, there is not reported evidence in literature of circulating cardiac myxoma cells (CCMCs), in this work we have studied a series of 10 sporadic cardiac tumors using a peripheral blood culture method^9–15^—recognized as effective for the search for circulating tumor cells.

To date, most studies on CM have focused on the characterization of tumor cells in situ, therefore a next step is the profiling of the liquid biopsy to produce valuable orthogonal data for the definition of this ambiguous pathology.

The multiparametric approach proposed here in the CM integrates multiple tumors and stromal phenotypes—such as endothelial cells—based on blood to produce an informative profile of the tumor that can be acquired presurgical. To test the technical feasibility of such an approach, we undertook a pilot study using blood samples from a small cohort of CM patients. Each blood sample was analyzed for the presence of CTCs, and CECs and compared with data obtained from PDX models, and these data were used to generate an overview profile of multiparameter liquid biopsy specific for patients with CM.

## Materials and methods

### Study design and population

Study design has been carried out according to local and international institutional guidelines and has received formal approval by our referred Ethic Committee, Bioethics Expert, and University Hospital “Mater Domini.” (CHARActerization of Circulating Tumor cells and Expansion) CHARACTEX Project Number: 2013.34 including the subjects belonging to the healthy cohort as a control group enrolled at Department of Health Sciences of Magna Graecia University. In particular, the inclusion criteria adopted to enroll volunteers were:

#### Tumor patients

- Caucasian race.
- Patients with diagnosis of cardiac myxoma.
- Age between 18 and 85 years.
- Effective contraceptive methods in cases where there is the possibility of conception.
- Written informed consent.

#### Healthy subjects or control

- Caucasian race.
- Non-smoking healthy male and female adults.
- Age between 18 and 85 years.
- State of good health supported by the most relevant clinical and biochemical parameters.
- Effective contraceptive methods used in cases where there is the possibility of conception.
- Written informed consent.

Moreover, cardiac patients enrolled have signed informed consensus approval by our referred Ethic Committee, Bioethics Expert, and University Hospital “Mater Domini” with Cod. Prot. ENDO-FIRE study to enroll cardiac patients with atrial fibrillation and with Cod. Prot. ENXO-cardia study to enroll cardiac patients with heart dysfunction at Cardiovascular Institute, Magna Graecia University, Catanzaro, Italy.

### Tissue sample processing

**Figure 1:**
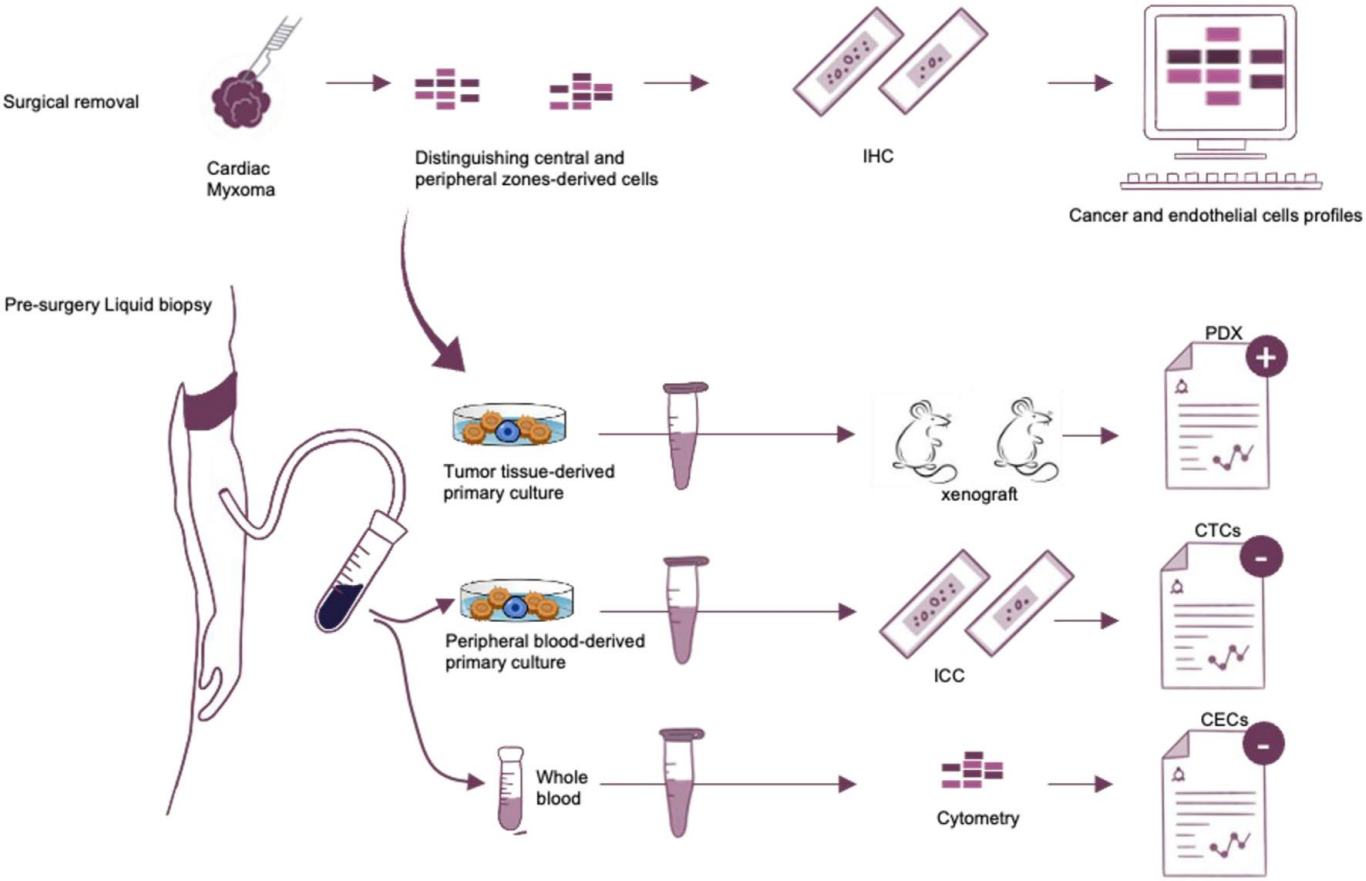
Experimental workflow. Cardiac myxoma (CM) tissue biopsies were processed pooled and stained distinguishing central and peripheral tumor regions. Primary cell cultures enriched for cancer cells were obtained from tumor tissue and peripheral blood of CM patients. Phenotypic profile of cancer and endothelial cells were analyzed by immunological testing in tissue, cell cultures and PDXs. *PDX* patient derived xenograft, *IHC* immunohistochemistry, *CTCs* circulating tumor cells, *ICC* immunocytochemistry, *CECs* circulating endothelial cells.

We collected tumor samples from 10 patients with Cardiac Myxoma who had not received previous therapy. Identifiers of patients were reassigned to protect anonymity and blood sample was collect before the surgery removal. Eligible patients were at least 18 years of age and had received a diagnosis of CM based on preoperative investigations (in the 70% of the patients the suspicions of CM the diagnosis was formulate at a routinary echocardiography examination) and postoperative histologic analysis. The cohort was representative of a population of patients with CM who were eligible for curative resection. Histologic data were confirmed on central review by pathologist in agreement with 2015 WHO Classification of Tumors of the Heart and Pericardium (Details regarding the study design are provided in Fig. 1. To assess intratumor heterogeneity, samples of at least two tumor regions that were separated by a margin of 0.5–1 mm (depending on the size of the tumor) had to be available for study. All the patients provided written informed consent. The clinical characteristics of the patients and the study criteria are provided in Table S1. We used the immunohistochemical testing and western blot analysis to perform cellular heterogeneity on multiple regions collected from each tumor. We characterized 20 tumor regions (all primary tumor regions) and 10 matched samples derived from whole blood. Orthogonal validation was performed on correspondent primary cell cultures from tissue and from blood according to previously published protocol detailed in Supplementary Materials and Methods.

Primary myxoma cell culture collected were characterized by immunocytochemical and cytometry testing and subcutaneously injected in Female Fox-nude mice, 5 weeks aged, according to the institutional guidelines for animal care approved by Magna Graecia Institutional Review Boards on Animal Use and Welfare. All animal experimental procedures were performed according to the Guide for the Care and Use of Laboratory Animals from directive 2010/63/EU of the European Parliament.

### Blood sample processing

Peripheral blood (5 ml) has been collected from each patient into tubes containing EDTA and centrifuged into Ficoll-Paque Plus (GE Healthcare) to reduce the haematological cells. According to the heterogeneous size and relative cell density of cancer cells, the suspension enriched for cancer cells has been isolated from cell layer comprised between 1080–1090 (g/m) density gradient values, as previously demonstrated^9^. At the end of 14 days in order to characterize the circulating non-hematological cells in blood-derived cultures on chamber slides, the cells cultivated on slides were fixed with a 4% paraformaldehyde solution and stored at 4 C° after 2 weeks. Successively, slides were stained following a standard H&E staining protocol^9^. Briefly, slides were treated for heat-induced epitope retrieval and after staining with primary antibody, in particular CD31 and Calretinin. The immunocytochemical reactions were visualized with 3,3′‐diaminobenzidine (DAB) solution and the nuclei were counterstained with hematoxylin. After the phase of dehydratation through a series of graded alcohols the slides were evaluated by two pathologists experienced in cancer disease that interpreted each case independently before arriving at a consensus reference diagnosis. For the immunofluorescence assay the cells were fixed and blocked and then incubated with the following primary antibodies: Calretinin, c-Kit and CD31 visualized by Alexa Fluor 488-conjugated anti-mouse IgG. The nuclei were counterstained with DAPI. Moreover, for cell cycle analysis the cells have been washed and fixed with 70% ethanol at − 20 °C overnight. Cell cycle phases distribution was performed using the CycleTEST plus DNA reagent Kit and the analysis was performed with ModFit LT software (entire procedures, type of antibody and dilution are detailed in Supplementary Materials and Methods).

For circulating endothelial cells analysis, a total volume of 10 ml was collected. For each sample, 20 × 10^6^ leukocytes were processed within 4 h from blood collection. The pellet of each sample was added to the lyophilized cocktail of reagents, previously re-hydrated by the addition of 100 µl of Stain Buffer (BD Biosciences); 1 µM Syto16 was finally added to each tube. All the samples were incubated, centrifuged and re-suspended in FACSFlow (BD Biosciences). Flow cytometry acquisition was of 2–4 × 10^6^ events/sample with lymph-monocyte morphology by (FACSAria, BD Biosciences) Endothelial cells were identified as already described^10^; cells were plotted using dot-plot bi-exponential display in order to ensure correct gate placement. To assess non-specific fluorescence, were used both fluorescence minus one and isotype controls in combination with all the remaining surface reagents present in the panel. Endothelial cells numbers were calculated by a dual-platform counting method using the lymphocyte subset as reference population as previously reported^10^. Flow cytometry data was analyzed by FACSDiva v. 6.1.3, and FACSuite v1.05 (BD) and CyTOF Software 6.7(CyTOF 6.7) single-cell data software (detailed procedure are reported in Supplementary Materials and Methods).

### Statistical analysis

The quantitative data are presented as mean ± standard error. Data have been analyzed by using Student’s 2-tailed *t* test to identify statistically significant differences between groups. Results are reported as mean ± standard error. The significance level was set at p < 0.05. Comparison between patients and control group was performed using Mann–Whitney and Kolmogorov–Smirnov tests with a valid statistical significance of p < 0.05 Sub-groups were compared using the *t*-test (for continuous variable). All statistical analyses were performed using MedCalc for Windows, version 18 (MedCalc Software, MariaKerke, Belgium).

### Ethics approval and consent to participate

The study design has been carried out according to local and international institutional guidelines and has received formal approval by our referred Ethic Committee, Bioethics Expert, and University Hospital “Mater Domini.” CHARACTEX Project Number: 2013.34 including the sub-jects belonging to the healthy cohort as a control group enrolled at the Department of Experimental and Clinical Medicine of Magna Graecia University. Moreover, cardiac patients enrolled have signed informed consensus approval by our referred Ethic Committee, Bioethics Expert, and University Hospital “Mater Domini” with Cod. Prot. ENDO-FIRE study to enroll cardiac patients with atrial fibrillation and with Cod. Prot. ENXO-cardiac study to enroll cardiac patients with heart dysfunction at Cardiovascular Institute, Magna Graecia University, Catanzaro, Italy; and URT-CNR, Magna Graecia University.

## Results

### Demographic and baseline clinical characteristics of cardiac myxoma patients

The predominant clinical manifestations in the cohort of patients with cardiac tumors were resumed in Supplementary Table S1 were in 13% with a cardiovascular symptom (hypertension, diabetes, inflammation, atrial fibrillation). In the 95% cases, CM was diagnosed accidentally, during regular echocardiography control. Echocardiography appearance of tumors had a smooth surface 53% and the remaining villous surface.

### Cancer and endothelial cell characterization in tumor regions of cardiac myxoma

**Figure 2:**
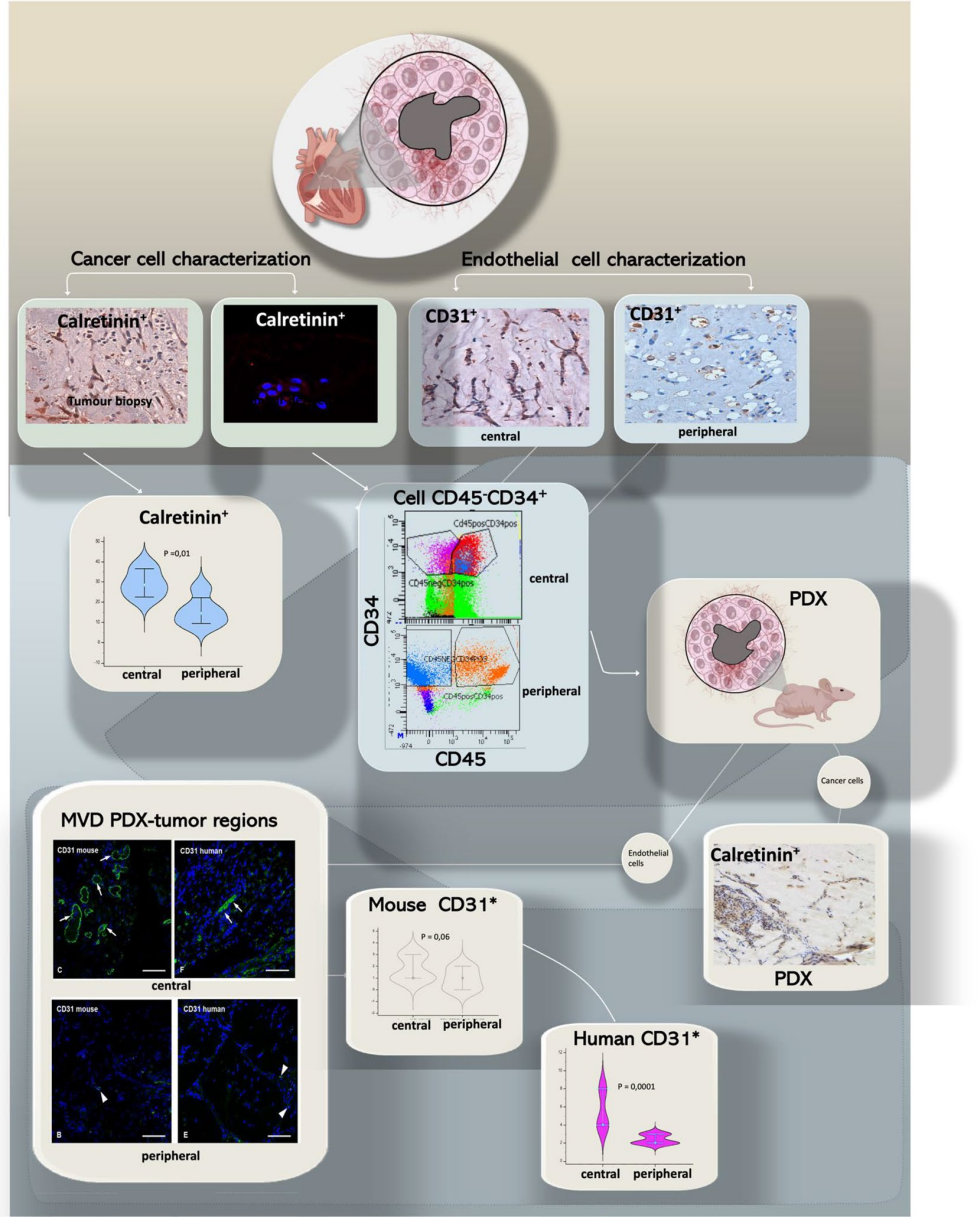
Cancer and endothelial cell characterization in tumor biopsy. Tumor biopsy was processed to distinguish central and peripheral regions of the cardiac myxoma. Immunohistochemical analysis for the expression of calretinin shown a different distribution of calretinin positivity cells prevalently distributed in the central region of the tumors (P = 0.01) and for CD31 expression (P = 0.001). Primary culture tumor region derived, preserved calretinin positive cells and showing at FACS analysis a different distribution of cell CD45 negative CD34 positive (P = 0.008). PDXs derived distinguishing central and peripheral region confirmed the prevalent density of vessel-forming units in the central area of cardiac myxoma.

All 10 tumors exhibited the typical histology of cardiac atrial myxoma. To characterize tumour cells population of CM to different levels of differentiation, markers of neural derivation such as Calretinin (p = 0.01) and CD31 (p = 0.00) routinely used in diagnostic of CM, were analysed and a prevalence in the central region for both antigen expression was recognized (Fig. 2). Moreover, CMs were processed and disaggregated into single cell suspensions perform primary tumour-derived cultures and patient-derived xenografts (PDXs).

Immunofluorescence on primary cultured cells was performed to analysed the expression of Plexin A2 (that characterizes Cardiac neural crests) and Semaphorin 3C (normally associated to Plexin A2) in agreement with the known role of cardiac neural crest cells for proper development of the cardiovascular system^3^. Moreover, alpha-smooth muscle actin (α-SMA), normally expressed in differentiating cardiomyocytes, was analysed^16,17^ 40% of myxoma cells expressed α SMA, 67% Plexin A2, 55% Semaphorin 3C, and 30% Calretinin (Supplementary Fig. 1). The endothelial cells in the primary tumour derived culture were characterized by FACS analysis. The primary cell culture central region derived was enriched for CD34^bright^ (7% ± 2) rather than peripheral (1% ± 0.2) one. In the central region of the tumor, the cells CD45^neg^CD34^bright^ co-expressing CD309^pos^ (73% ± 22) indicated the prevalent population of progenitor cells committed in endothelial sense. Mature endothelial CD45^neg^CD146^pos^ cells were prevalently concentrated in the central zone (p < 0.005). Moreover, after short-time in vitro expansion, heterogeneous CM cells were transplanted (Fig. 2), in immunodeficient-mouse to obtain PDXs. CD31human and CD31mouse antibodies, for discriminating angiogenesis/vasculogenesis processes in PDXs vessel-distribution, showed lower vessel-density in peripheral region (Fig. 2). PDX central region were enriched for CD31^mouse^ (63 ± 9%) expression (p = 0.06) with vessel density rate (55 ± 7%) indicating angiogenetic process starting from preexisting mouse vessels. In addition, CD31^human^ (37 ± 2%) expression (p = 0.0001) and vessel density rate (15 ± 2%) were indicative of vasculogenesis, starting from human endothelial progenitor cells, in agree with endothelial cell hierarchy observed before the transplantation.

### Cancer and endothelial profiles in CM liquid biopsy

**Figure 3:**
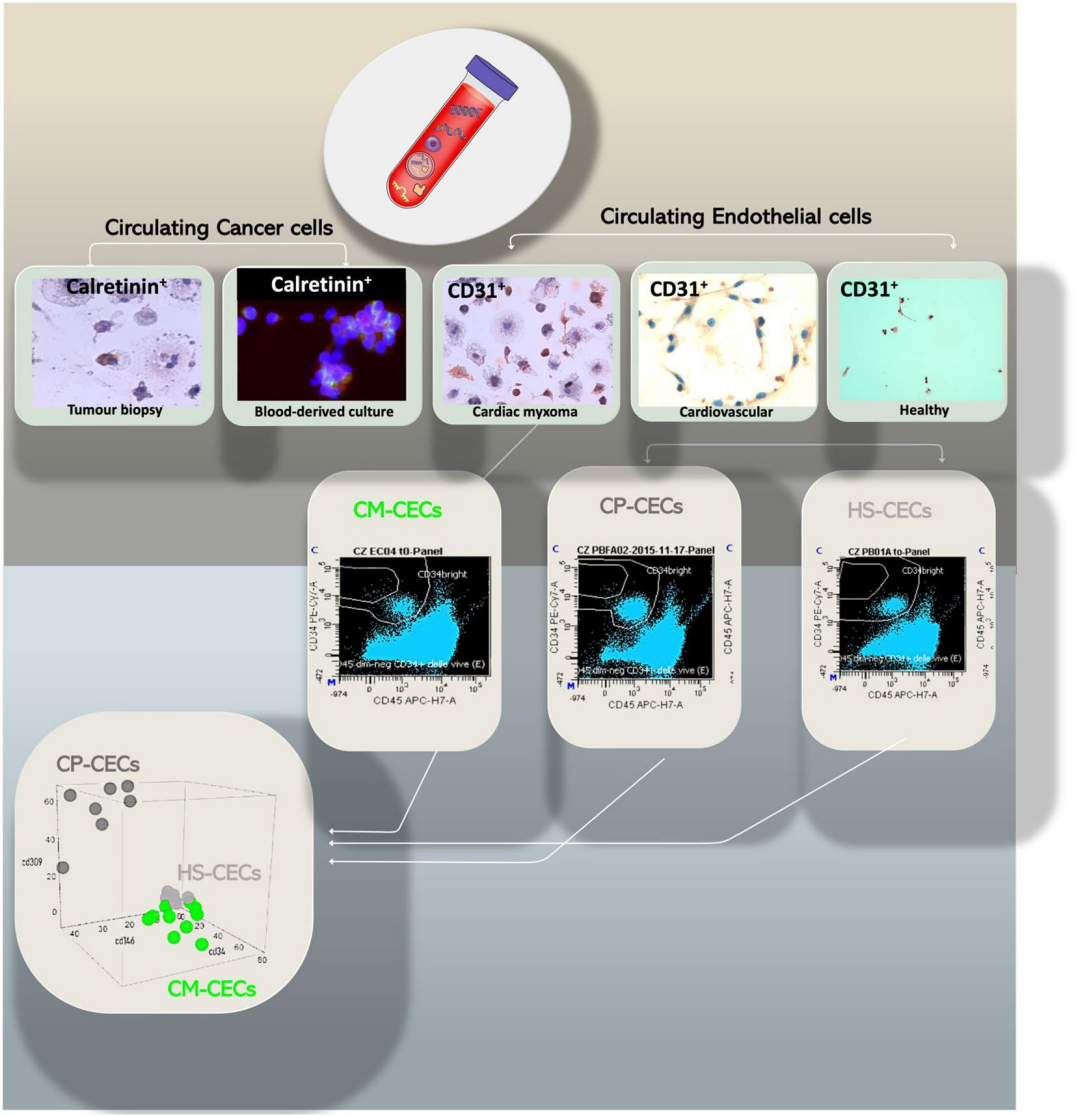
Cancer and endothelial profiles in cardiac myxoma liquid biopsy. Immunocytochemistry analysis shows the presence of cells calretinin positive (also visible at immunofluorescence assay) and CD31 positive. Cells CD31 positive are recognizable in blood-derived cultures of cardiovascular patients and healthy subject as expression of vessel forming unit cells prevalently presented in the peripheral blood of cardiovascular rather that CM patients.

**Figure 4:**
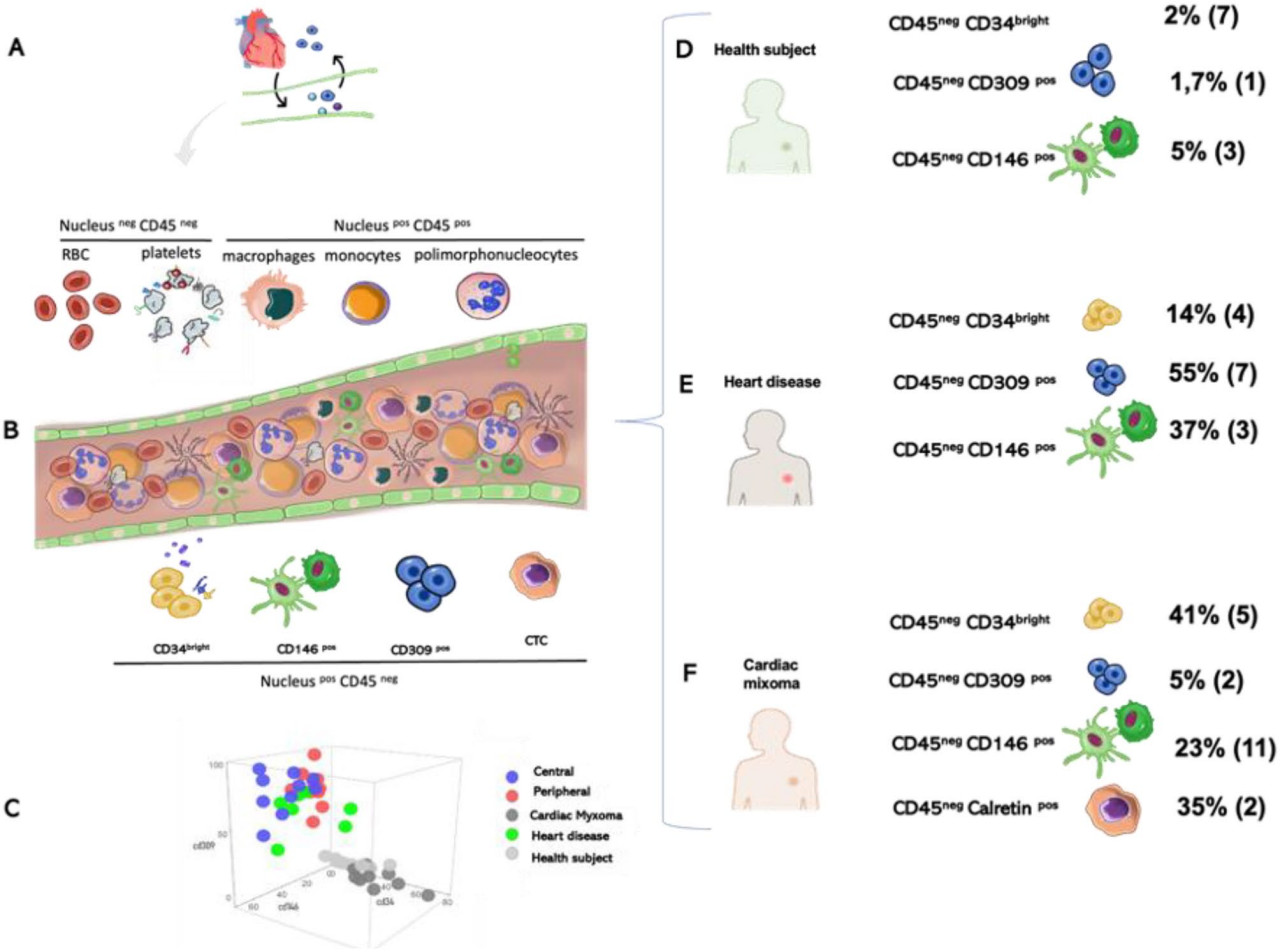
Visual graphical illustration of phenogroup blood-based cellular biomarkers. (**A**) The exchange of mediators and cells between the heart and the systemic circulation affects the type of non-hematological cells within the peripheral vessel (**B**) characterizing the cell type observed by blood collection (**C**) the graph shows the distribution of the circulating endothelial cells subsets in function of the subset of endothelial cells isolated from different region of tissue biopsy. (**D**) In the healthy subject the condition of heart health determines a profile mainly characterized by the CD34 bright cells which are part of the progenitor compartment with multiple differentiation abilities. In (**E**) the heart disease patients the pathological condition of the heart determines a greater recruitment of committed progenitors in the endothelial sense probably to support the increased demand of blood flow. (**F**) In cardiac myxoma the heart is the site of a proliferative lesion which autonomously contributes to the non-hematological component of the blood, evidencing a tissue remodeling which also includes the intratumor vascular network.

Peripheral blood samples were in parallel processed by Malara et al.^9–15^ and Lanuti et al.^10,18^ protocols to detect respectively circulating tumor and endothelial cells. Circulating cells Calretinin^pos^ was found in the cytological preparation obtained from blood analyzed with immunocytochemistry and by fluorescence. The mean value of percentage of circulating Calretinin^pos^ cells of CM cohort was of 35.1 ± 2%. The immunocytochemistry analysis for the expression of CD31 showed a percentage of 8 ± 0.2% cells positive (Fig. 3). Moreover, FACS analysis performed on whole blood sample comparing three different cohorts, cardiac myxoma, cardiovascular patients and healthy subjects, showed cells CD45^neg^ CD34^bright^ (41 ± 4.8%) in CM cohort, (14 ± 4%) in cardiac patients and (2 ± 7%) in healthy subjects (Fig. 4). The population of progenitor cells, CD45^neg^ CD34^bright^ cells and CD309^pos^ were (5 ± 2%) in CM cohort, 5 ± 3% in healthy and 55 ± 7% for cardiac patients respectively. The population of CECs, CD45^neg^CD146^pos^ cells, showed 37 ± 3% in cardiac patients’ cohort, 5 ± 3% in healthy subjects and 23 ± 11% in CM patients. Significant statistical difference (p = 0.001) was observed between healthy subjects and CM patients for the percentage of CD45^neg^ cells (p = 0.005).

## Discussion

Although disseminations of myxoma have been described by embolic mechanisms as metastatic localization to the brain^4^, circulating tumour cells of this type of tumour have never been described. At the base of this, probably, there are contradictory works concerning its presumed metastatic power. Today, we can affirm for CM, as for other types of tumours, i.e. glioblastoma, despite the rarity of metastatic events, corresponding circulating tumour cells are recognizable in the peripheral blood^14,19^. In CM the finding of disseminated cell population could be interpreted as an epiphenomenon linked to its anatomical location. Its development inside the cardiac cavity exposes CM to a mechanical solicitation favouring casual cells releasing into the circulation. Proof of this is that the embolic event was described less rarely than the metastatic event^20^. In the cohort of CM patients enrolled in this study, corresponding CTCs, cannot be considered as the consequence of the mechanical event alone. Our data sustained that all parts of the tumour could contribute to disseminate cancer cells. The disseminated cancer cells preserved a central tumour-region derived phenotype, also justify by greater presence of vessels in this part of the tumour. In fact, the central region derived PDXs were characterized by tumorigenic and vasculogenesis findings, confirming what has already been previously proven on myxoma histogenesis^8,21^ and suggesting the hypothesis of a perivascular cancer stem niche. Regarding the interpretation of the mesenchymal multiple differentiation capacity of CM cells, however, it is interesting to remember that these cells could also originate from elements of the c-kit positive cardiac neural crests^22^.

The markers we had chosen in this paper were or used in histopathological diagnosis of CM, like as Calretinin, CD31 and CD34 or linked to a hypothesis of derivation of CM from cardiac neural crests (Plexin A2 and Semaphorin 3C). The hypothesis of the existence of a pool of cardiac stem cells c-kit positive, derived from cardiac neural crests is experimentally sustained from various authors^23,24^.

Finally, comparative analysis of endothelial cell hierarchy between liquid and tissue biopsy in CM intrigued the paradigm of the interplay heart/bone marrow physiologically involved in case of reduction of the partial tension of oxygen at the cardiac tissue to supply endothelial progenitor cells. Our results demonstrated that the cardiac myxoma the cellular architecture of central area was enriched by stem cell with mesenchymal address (CD45^neg^ CD34^bright^), as reported in other tumors^13,25^. Furthermore, the endothelial progenitor CD34^bright^ CD309^pos^ cells were able to induce both vasculogenesis and angiogenic events^10,11^, as evidence by CD31^human^/CD31^mouse^-expressing endothelial cell rate in PDXs. The autonomy to forming vessel in CMs was further sustain by the observed low number of EPCs (CD45^neg^ CD34^bright^ CD309^pos^) in the peripheral blood analyzed. However, cardiac patient’s cohort displayed an increased circulating pattern for both CECs and EPCs caused by conditions that determined tissue sufferance and variations of local oxygen levels, as hypertension and atrial fibrillation^10,11^. The prevalence of EPC in the peripheral blood of heart disease cohort is the same observed within the central region of the CM (Fig. 4C).

## Conclusion

**Figure 5:**
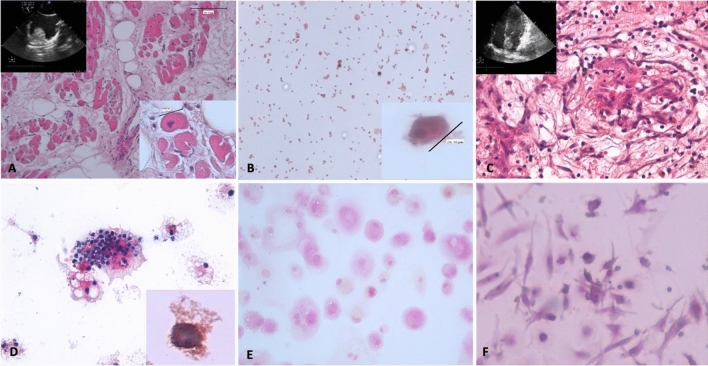
Blood- based cellular biomarkers of other cardiac tumors. (**A**) Histopathological section (magnification 20 ×) of the tumor stained with hematoxylin and eosin is reported of a case of atrial villous angiomyolipoma, diagnosed in a 39-year-old male. In the left top of the image its echocardiographic picture shows the mass arising from the atrial wall. In the right bottom of the image, a particular of a tumor cell (magnification 40 ×). In (**B**) his cultured circulating cells specimen (magnification 10 ×) shows in the right bottom of the image, cytomorphological feature like tumor cell in primary lesion (magnification 40 ×). (**C**) Histopathological section (magnification 20 ×) of a case of atrial myxoma, hematoxylin and eosin stained, diagnosed in a 60-year-old male during an echocardiographic control. Echocardiographic picture is reported in the left top of the image and in (**D**) cultured circulating cells (magnification 20 ×), in the right bottom of the image a particular of a calretinin positive cell (magnification 40 ×). (**E**) and (**F**) show cultured circulating cells isolated from blood sample of a 20-year-old female angiosarcoma patient and a case of 69-year-old male fibroelastoma, respectively (magnification 20 ×).

Taken together the blood-based profile of the cellular biomarkers examined suggest a specific profile for cardiac myxoma in respect to cardiovascular patients in terms of presence of tumor cells disseminated from the primary tumor and for a particular subset of endothelial cells the configured different functional condition of the vessel-forming unit cells within the heart. The phenogroups here profiled could be useful to differentiate the cardiac myxoma in which the echocardiography is not able to differentiate a intracardiac thrombus from a intracardiac tumors and clarify within the cardiac tumors different histology (Fig. 5).

### Supplementary Information

Supplementary Figures.

Supplementary Information.

Supplementary Table S1.

## Data Availability

All supporting data are included in the manuscript and supplemental files. Additional data are available upon reasonable request to corresponding author.
