# Supplementary material for: Distinctive phenogroup to differentiate diagnosis of cardiac myxoma vs cardiovascular disease examining blood-based circulating cell biomarkers
Source: Sci Rep. 2023 Nov 21;13:20357. doi: 10.1038/s41598-023-47639-y (PMC10663517; doi:10.1038/s41598-023-47639-y)
Supplement: Supplementary file 1 — Supplementary Figures. [file 41598_2023_47639_MOESM1_ESM.docx]

**Supplementary Figures**

**Figure S1**
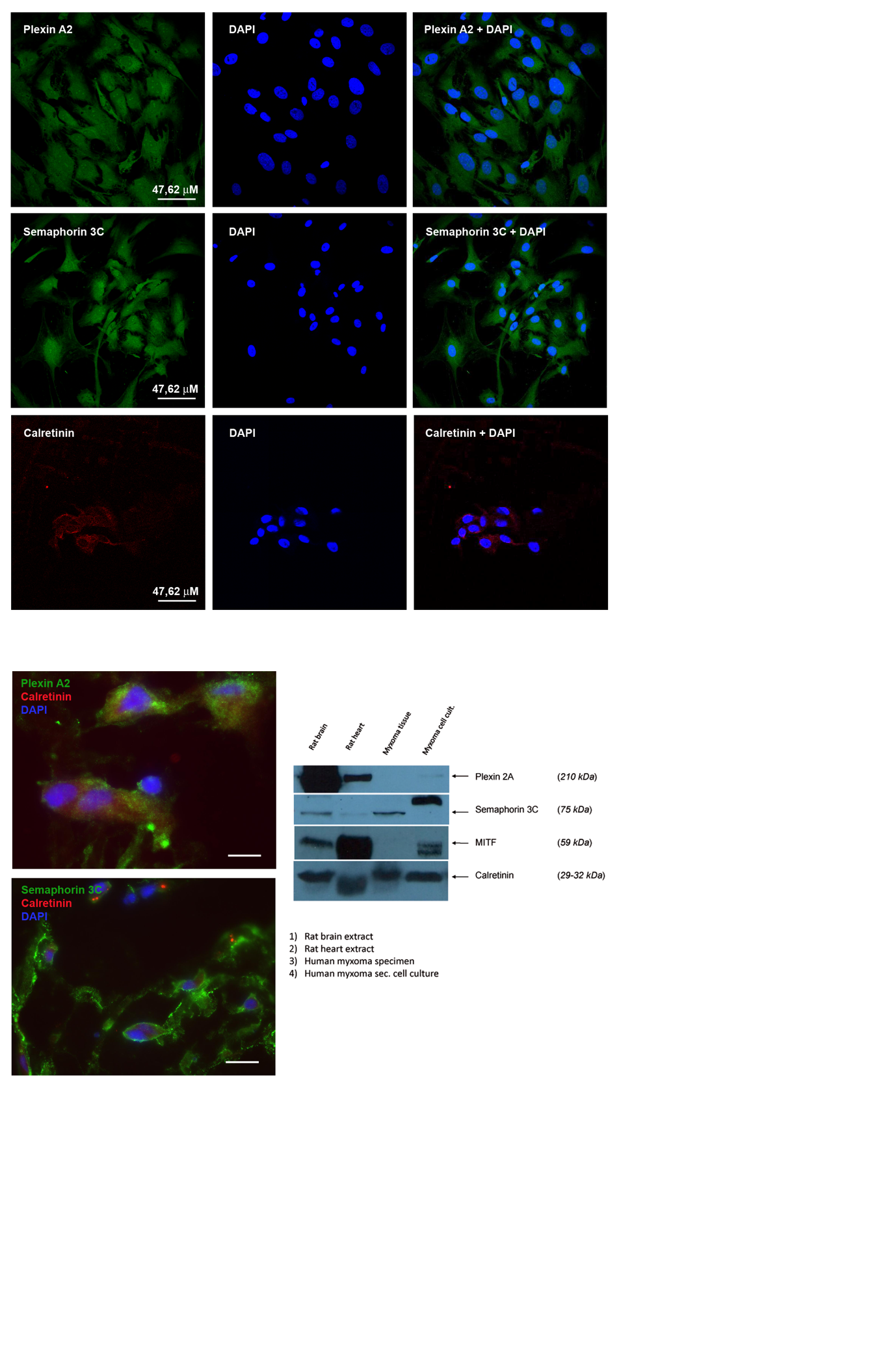


Representative images of immunofluorescence analysis on Human Cardiac myxoma tissue of Plexin A2, Semaphorin 3C/ and Calretinin. Nucleus in blue are stained with DAPI Double immunofluorescence analysis of Plexin A2/Calretinin, Semaphorin 3C/Calretinin and α- SMA on primary cell culture Cardiac myxoma tumour tissue are reported.

The image of the Western blot analysis shows comparative expression for the proteins, Plexin A2, Semaphorin, MITF protein (Microphthalmia-associated Transcription Factor) and calretinin in total cellular extract of different tissues, brain and heart in rat and human.

**Unprocessed western blot from Figure S1**


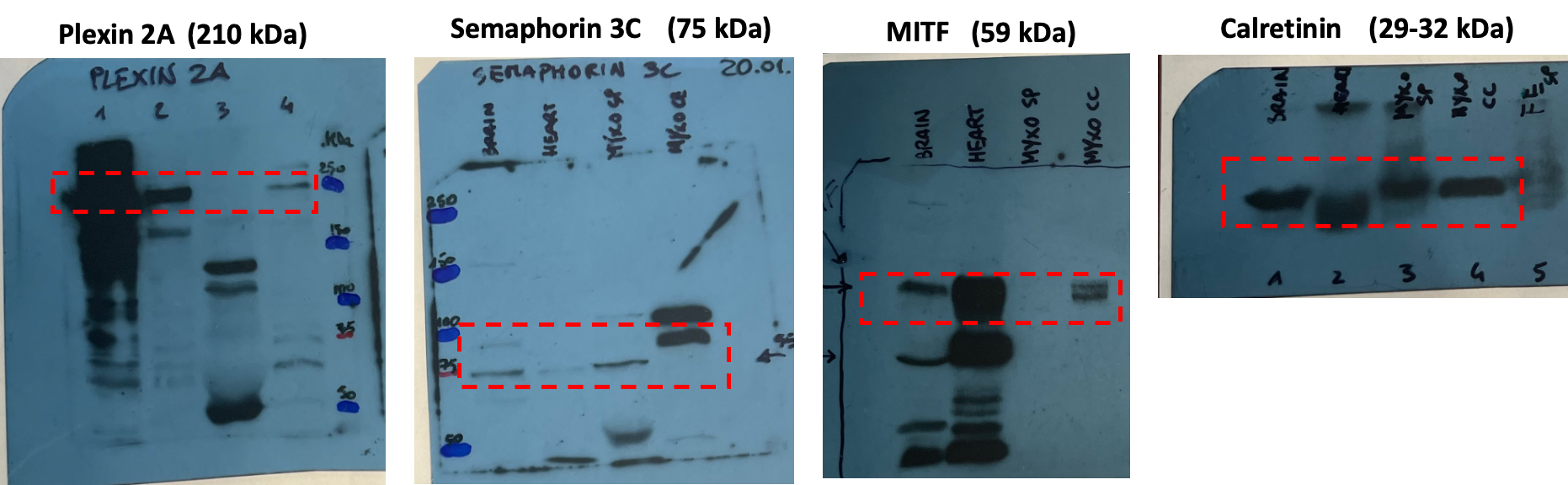


Unprocessed images of all gels and blots

**Figure S2**


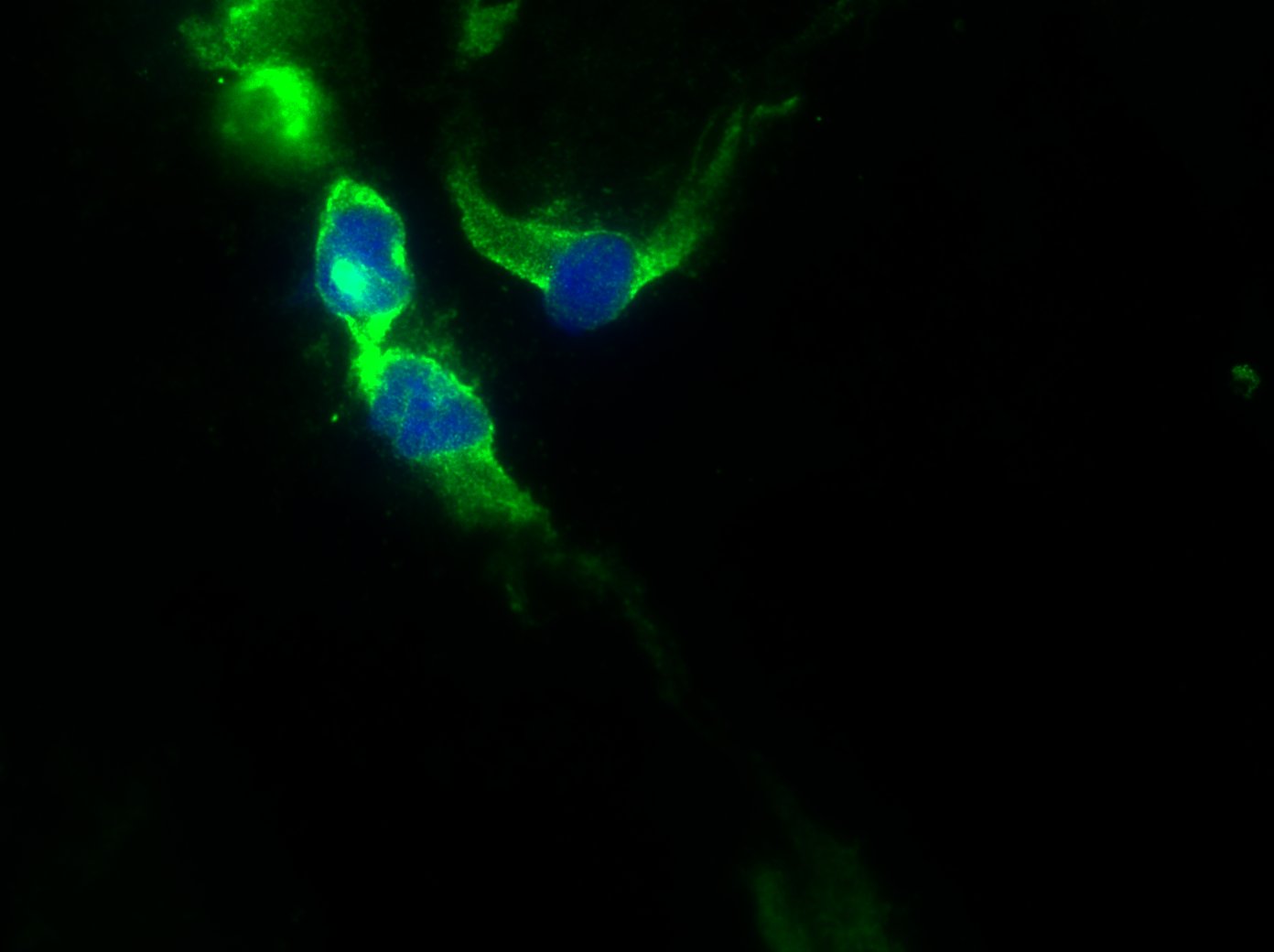


Plexin A2

Representative image of immunofluorescence analysis on circualting Human Cardiac myxoma of Plexin A2
