# Supplementary material for: Distinctive phenogroup to differentiate diagnosis of cardiac myxoma vs cardiovascular disease examining blood-based circulating cell biomarkers
Source: Sci Rep. 2023 Nov 21;13:20357. doi: 10.1038/s41598-023-47639-y (PMC10663517; doi:10.1038/s41598-023-47639-y)
Supplement: Supplementary file 2 — Supplementary Information. [file 41598_2023_47639_MOESM2_ESM.docx]

**Supplementary Materials and Methods**

**Patients Selection**

Patients with echocardiographic diagnosis of cardiac mass have been enrolled at S. Anna Hospital of Catanzaro. Informed consensus on general content of study methodology and objectives has been collected from each individual patient entering the study. In addition, study design has been carried out according to local and international institutional guidelines and has received formal approval by our referred Ethic Committee, Bioethics Expert, and University Hospital “Mater Domini.” CHARACTEX Project Number: 2013.34 including the subjects belonging to the healthy cohort as a control group enrolled at Department of Experimental and Clinical Medicine of Magna Graecia University. Moreover, cardiac patients enrolled have signed informed consensus approval by our referred Ethic Committee, Bioethics Expert, and University Hospital “Mater Domini” with Cod. Prot. ENDO-FIRE study to enrol cardiac patients with atrial fibrillation and with Cod. Prot. ENXO-cardial study to enrol cardiac patients with heart dysfunction at Cardiovascular Institute, Magna Graecia University, Catanzaro, Italy; and URT-CNR, Magna Graecia University. Clinical Pathological findings of all patients and healthy subjects enrolled are detailed in Supplementary Table S1.

The inclusion criteria for our study were as follows:

Cardiac Tumour patients Cohort (CHARACTEX Project Number: 2013.34)

- Caucasian race
- Patients with confirmed diagnosis of space occupying lesions or masses in the heart
- Age between 18 and 85 years
- Effective contraceptive methods used by both men and women in cases where there is the possibility of conception.
- Written informed consent.

Healthy Subjects Cohort (CHARACTEX Project Number: 2013.34)

- Caucasian race
- Male and female healthy and non-smoking adults
- Age between 18 and 85 years
- State of good health supported by the most relevant clinical and biochemical parameters (for example: normal systolic and diastolic blood pressure values, normal blood count and blood values).
- Effective contraceptive methods used in cases where there is the possibility of conception.
- Written informed consent

Cardiac Patients Cohort

- Caucasian race
- Male and female adults
- Age between 18 and 85 years
- Diagnosis of NSTEMI (Cod. Prot. ENXO-cardial study)
- Diagnosis of AF (Cod. Prot. ENDO-FIRE study)
- Written informed consent

**Tissue sample processing**

Tumour tissues samples, have been collected from patients who underwent therapeutic radical cardiac tumour resection. Two independent pathologists have confirmed the histological diagnosis of cancer in all cases involved. The cancer disease was classify in agreement with 2015 WHO Classification of Tumors of the Heart and Pericardium.

**Design of tumour tissue zones.** Myxoid and other type of human cardiac tumor tissue samples were obtained from patients undergoing heart surgery. In particular, cardiac myxoma specimens were sliced from apex to base at 3–4-mm intervals. Cancer involving the central zone was defined as cancer foci involving the expected location of the central zone at the base of the tumour implantation. Correlation between pathologic features and echocardiography imaging findings was performed in consensus by three of the authors (S.G., D.M., G.D.). The central zone outlined on the whole-mount pathologic maps was evaluated in conjunction with the echocardiography to establish the location and visibility of the central zone on images by using anatomic landmarks of atrial and ventricles.

**Immunohistochemical analysis.** For confocal microscopy, sections were deparaffinized before endogenous peroxidase quenching and heat-induced epitope retrieval. To characterize the distribution pattern of staining to detect c-kit (CD117) (polyclonal rabbit, 1:400; Dako), Carletin (monoclonal mouse, 1:100; Leica), CD31 (monoclonal mouse, 1:40; Dako) were performed. After permeabilization and blocking with 100 µl 0.5% saponin and 10% BSA in PBS 1× for 30 min, sections were incubated (1–2 h) with primary reagents in the same buffer used for permeabilization. Slides were extensively washed with PBS 1×, and then the cells were sequentially incubated with streptavidin and biotin using reagents of the avidin/biotin blocking kit from Vector. The staining procedure was repeated three times [1-3].

**Primary cultures tumor- derived.** Primary myxoma cell cultures were obtained ex vivo from human tumors. Cells were extracted by both enzymatic dissociation with trypsin (Sigma-Aldrich, St. Louis, MO) and outgrowth methods. However, myxoma cell cultures obtained by outgrowth method display greater proliferation rates and defined morphology. Myxoma cells were grown on gelatin-coated plates in RPMI medium with 20% fetal bovine serum at 37°C in a 5% CO2 atmosphere. When the cell cultures reached 70-80% confluency around the small pieces of tissue, between 7 and 14 days, the myxoma cells were sub-cultivated as the first cell passage. The cells were seeded approximately at 4000 cell/cm2. When the cells became 80-90% confluent, they were sub-cultured until passage 5. At each passage, the cells were harvested by enzymatic procedure. For each experiment, cells derived from at least three different individuals at passages 2-5 were used. After morphological examination, cells were used for subsequent analysis [2-3].

**Immuno*cyto*fluorescence analysis.** Myxoma cells were cultured on cover slip. After two wash in PBS, cells were fixed with 0.3% gluteraldehyde (Sigma Aldrich) for 10 min at 37 °C and then washed in PBS. Cells were permeabilized in 0.2% Triton X-100, blocked in 10% FBS in PBS for 30 min and then incubated with the following primary antibodies: c-Kit (CD117-Phycoerythrin RD System LMW02 1:50), Calretinin (Leica Biosystems, NCL-L-CALRET-566, 1:200). Semaphorin 3C (Santa Cruz Biotech, sc-27796; 1:100), Plexin A2 (R&D Systems, AF5486, 1:100) CD31 (monoclonal Mouse IgG1 Clone # 9G11 RD) Carletin (monoclonal mouse, 1:100; Leica) CD34 (monoclonal mouse 1:100 AC136 Miltenyi biotec) Concanavalin A (polyclonal goat1:100 AP21430HR-N). System followed by the following species-specific fluorescence-conjugated secondary antibodies. In particular, Alexa Fluor 488-conjugated anti-goat IgG and Alexa Fluor 546-conjugated anti-mouse IgG. Slides were counterstained with DAPI (Santa Cruz Biotechnology; 2 μg/mL), mounted using an antifade mounting medium (Life Technologies), and observed with a Laser Confocal Scanning Microscopy (SP2 LSCM, Leica Microsystems).

**Cytometry analysis.** From each tissue were obtained cellular suspensions processed for cytometric analysis. Human cardiac myxoma tumor tissues, distinguishing central and peripheral zones, were excised and suspensions of cells were analysed for the presence of endothelial cells. In order to preserve high level of standardization, lyophilized reagents (Becton Dickinson, San Jose, CA, USA; Lyotube, Custom cat # 623920) for the panel and the related control tube were used, as previously reported [9]. The pellet of cells were processed following the cytometry protocol detailed in the section *circualting endothelial cells*, reported below.

**Patient-derived xenografts (PDXs).** Female Fox-nude mice, 5 weeks aged, were cared according to the institutional guidelines for animal care approved by Magna Graecia Institutional Review Boards on Animal Use and Welfare. All animal experimental procedures were performed according to the Guide for the Care and Use of Laboratory Animals from directive 2010/63/EU of the European Parliament. All animals received humane care, and all efforts were made to minimize animal suffering. Mice were housed under controlled conditions of 25°C, 50% relative humidity and a 12-hr light (6:00 –18:00) and 12-hr dark cycle, with water and food (containing 18 % protein) available ad libitum. Primary myxoma cell culture were collected and subcutaneously injected, without anaesthesia, at the left side of each mouse. 1.5 × 105 tumour cells from each tumor zones, central and peripheral, were suspended in 100 µl of matrigel (BD Biosciences) and injected [3]. At 80 day from scarified animals were collected organs and tissues. Animals were euthanized by pentobarbital over dosage Tumor tissues excised by 3 mice (2 from central and 1 from peripheral group) and by 5 control mice, were analyzed. Tissues were frozen and then sectioned (thick 8 μm) on slides.

**Immunohistofluorescence analysis.** Human myxoma specimens stored at −80 °C were mounted on a cryostat freezing stage to obtain a series of sections of 8 μm appropriate for double immunofluorescence protocol. For this part, sections of the same area/slide for a total of 3 slides/myxoma were used. After permeabilization in 0.2% Triton X-100, and blocking in 10% FBS in PBS for 30 min, sections were incubated overnight at 4°C with primary antibody in the same buffer used for permeabilization. Slides were extensively washed with 1× PBS, then incubated with secondary antibody (Alexa Fluor 488-conjugated anti-goat; 1:400; Santa Cruz Biotechnology) and washed again. In particular, the expression of CD31 was analysed by using anti human CD31- PECAM-Fluorescein Monoclonal mouse RD System and anti mouse CD31/PECAM-1 Mouse Alexa Fluor® 488-conjugated RD System. Sections were counterstained with DAPI (Santa Cruz Biotechnology; 2 μg/mL), mounted using an antifade mounting medium (Life Technologies) and observed with a Laser Confocal Scanning Microscopy (SP2 LSCM, Leica Microsystems).

**Blood sample processing**

**Circulating tumor cells**

Peripheral blood (5 ml) has been collected from each patient into tubes containing EDTA and centrifuged into Ficoll-Paque Plus (GE Healthcare) to separate cancer cells from the remaining cellular blood components. According to the heterogeneous size and relative cell density of cancer cells, the suspension enriched for cancer cells has been isolated from cell layer comprised between 1080÷1090 (g/m) density gradient values, as previously demonstrated by Malara et al [2-8]. After washing in Phosphate Buffered Saline (PBS) the cells were recovered in a medium promoting in vitro expansion for 14 days (short-term cultivation) both in plates and on Eppendorf Cell Imaging Cover glass. The medium composition was reported in Malara et al [1-8].

**Immuno*cyto*chemical analysis.** Blood-derived cell cultures were established and maintained on both plate and slides to perform morphological evaluation by Haematoxylin and Eosin (H&E) staining, parallel to the cytometric evaluation of cell cycle phases distribution. After 14 days of cultivations, cells on slides were fixed with a 4% paraformaldehyde solution and stored at 4 C°. Successively, slides were stained following a standard H&E staining protocol [9] and evaluated under a light microscope (Leica ICC50HD, Leica Microsystem, Milan, Italy). Immunocytochemistry was performed followed the protocol previously described [1,5,6]. Briefly, slides were submerged in either sodium citrate buffer or Tris–EDTA buffer for heat-induced epitope retrieval at 97 ◦C for 20 min. Staining with primary antibody, in particular CD31 (monoclonal mouse, 1:20; Dako) Calretinin (monoclonal mouse, 1:100; Leica) were used. Two pathologists experienced in cancer disease interpreted each case independently before arriving at a consensus reference diagnosis and a cytopathological score was assigned.

**Immuno*cyto*fluorescence analysis.** Blood-derived cultures performed on Eppendorf Cell Imaging Cover glass were used. After two wash in PBS, cells were fixed with 0.3% glutaraldehyde (Sigma Aldrich) for 10 min at 37 °C and then washed in PBS. Cells were blocked in 10% FBS in PBS for 30 min and then incubated with the following primary antibodies: c-Kit (CD117-Phycoerythrin RD System LMW02 1:50), Calretinin (Leica Biosystems, NCL-L-CALRET-566, 1:200). CD31 (monoclonal Mouse IgG1 Clone # 9G11 RD) visualized with Alexa Fluor 488-conjugated anti-mouse IgG. Slides were counterstained with DAPI mounted using an antifade mounting medium (Life Technologies), and observed with a Laser Confocal Scanning Microscopy (SP2 LSCM, Leica Microsystems) [4,5].

**Cell cycle analysis.** Blood-derived cultures performed on plates were used. Cells (3-7)x10^5^ have been washed with cold PBS and fixed with 70% ethanol at -20°C overnight. The cell pellets have been suspended in 500 µl PBS containing 2 mg/ml RNAse A (Sigma) and kept at 37°C for 60 minutes. Then the cell pellets have been stained as previously reported . DNA content has been analyzed by a FACScan (Becton and Dickinson San Jose, CA). Cell cycle phases distribution was performed using the Becton Dickinson kit: CycleTEST plus DNA reagent Kit, data acquisition using FACS Canto II (Becton Dickinson) and the analysis was performed with ModFit LT software (http://modfit-lt.software.informer.com/4.0/) [2-8].

**Circulating endothelial cells**

PB was drawn (21G needles) in EDTA (Ethylenediaminetetraacetic acid) Vacutainer tubes (BD Biosciences, San Jose, CA, USA, cat. 368861). The first harvested 3 mL tube was excluded from the analysis, to avoid the effects of the vascular damage caused by venepuncture. A total volume of 10 ml was collected. For each sample, 20 x 10^6^ leukocytes were processed as already described within 4 hours from material collection. The pellet of each sample was added to the lyophilized cocktail of reagents, previously re-hydrated by the addition of 100 µl of Stain Buffer (BD Biosciences); 1 µM Syto16 (Thermo Fisher Scientific, Eisai, Medipost - US) was finally added, as liquid drop-in, to each tube. Samples were incubated in the dark for 30 minutes at 4°C, washed with 2 mL of Stain Buffer (BD Biosciences), centrifuged (400 g, 10 min, room temperature), and re-suspended in 1.5 mL of FACSFlow (BD Biosciences). Flow cytometry acquisition was of 2-4 x 10^6^ events/sample with lymph-monocyte morphology by (FACSAria, BD Biosciences) at flow rate = 3. Standard Deviation of Electronic background Noise (SDEN) was assessed for all fluorescent parameters by Cytometer Setup and Tracking (CS&T) Beads (BD Biosciences). Data reproducibility were sustained and daily checked in real time by using the CS&T quality control Module (BD Biosciences) and further validated by the acquisition of Spherotech 8 peaks Rainbow Beads (Spherotec. Lake Forest, IL, USA). Endothelial cells were identified as already described [10]; to ensure correct gate placement, cells were plotted using dot-plot bi-exponential display. In order to assess non-specific fluorescence, both fluorescence minus one and isotype controls in combination with all the remaining surface reagents present in the panel were used. Endothelial cells numbers were calculated by a dual-platform counting method using the lymphocyte subset as reference population as previously reported. Flow cytometry data analyzed with FACSDiva v. 6.1.3, and FACSuite v1.05 (BD) and CyTOF Software 6.7(CyTOF 6.7) single-cell data software.

**Statistical analysis**

The quantitative data are presented as mean ± standard error. Data have been analyzed by using Student’s 2-tailed t test to identify statistically significant differences between groups. Results are reported as mean ± standard error. The significance level was set at p < 0.05. Comparison between patients and control group was performed using Mann-Whitney and Kolmogorov-Smirnov tests with a valid statistical significance of p <0.05 Sub-groups were compared using the T-test (for continuous variable). All statistical analyses were performed using MedCalc for Windows, version 18 (MedCalc Software, MariaKerke, Belgium).

**References**

1. Malara NM, Givigliano F, Trunzo V, Macrina L, Raso C, Amodio N, Aprigliano S, Minniti AM, Russo V, Roveda L, Coluccio ML, Fini M, Voci P, Prati U, Di Fabrizio E, Mollace V. In vitro expansion of tumour cells derived from blood and tumour tissue is useful to redefine personalized treatment in non-small cell lung cancer patients. J Biol Regul Homeost Agents. 2014 Oct-Dec;28(4):717-31. PMID: 25620181
2. Guadagno E, Borrelli G, Pignatiello S, Donato A, Presta I, Arcidiacono B, Malara N, Solari D, Somma T, Cappabianca P, Donato G, Del Basso De Caro M. Anti-Apoptotic and Anti-Oxidant Proteins in Glioblastomas: Immunohistochemical Expression of Beclin and DJ-1 and Its Correlation with Prognosis. Int J Mol Sci. 2019 Aug 20;20(16):4066. doi: 10.3390/ijms20164066. PMID: 31434323; PMCID: PMC6720904.
3. Malara, N., Trunzo, V., Foresta, U. et al. Ex-vivo characterization of circulating colon cancer cells distinguished in stem and differentiated subset provides useful biomarker for personalized metastatic risk assessment. J Transl Med 14, 133 (2016). https://doi.org/10.1186/s12967-016-0876-y,
4. Malara, N., Gentile, F., Coppedè, N. et al. Superhydrophobic lab-on-chip measures secretome protonation state and provides a personalized risk assessment of sporadic tumour. npj Precision Onc 2, 26 (2018). https://doi.org/10.1038/s41698-018-0069-7,
5. Coluccio, M.L.; Gentile, F.; Presta, I.; Donato, G.; Coppedè, N.; Valprapuram, I.; Mignogna, C.; Lavecchia, A.; Figuccia, F.; Garo, V.M.; Fabrizio, E.D.; Candeloro, P.; Viglietto, G.; Malara, N. Tailoring Chemometric Models on Blood-Derived Cultures Secretome to Assess Personalized Cancer Risk Score. Cancers 2020, 12, 1362. https://doi.org/10.3390/cancers12061362,
6. Malara, N., Guzzi, G., Mignogna, C., et al. Non-invasive real-time biopsy of intracranial lesions using short time expanded circulating tumor cells on glass slide: report of two cases. BMC Neurol. 16, 127 (2016)
7. Malara N, Innaro N, Mignogna C, Presta I, Pirrone KC, Donato A, Gangemi V, Sacco R, Mollace V, Donato G. (2018) La biopsia liquida nella diagnosi del carcinoma tiroideo indifferenziato. L'Endocrinologo Volume 19, Issue 5, pp 270–272
8. Guadagno E, Presta I, Maisano D, Donato A, Pirrone CK, Cardillo G, Corrado SD, Mignogna C, Mancuso T, Donato G, Del Basso De Caro M and Malara N. Role of Macrophages in Brain Tumor Growth and Progression. Int J Mol Sci. 2018 Mar 27;19(4). pii: E1005. doi: 10.3390/ijms19041005. Review
9. Paola Lanuti, Gianluca Rotta, Camillo Almici, Giuseppe Avvisati, Alfredo Budillon, Paolo Doretto, Natalia Malara, Mirella Marini, Arabella Neva, Pasquale Simeone, Elena Di Gennaro, Alessandra Leone, Alessandra Falda, Renato Tozzoli, Chiara Gregorj, Melania Di Cerbo, Valentina Trunzo, Vincenzo Mollace, Marco Marchisio, Sebastiano Miscia: Endothelial Progenitor Cells, Defined by the Simultaneous Surface Expression of VEGFR2 and CD133, are not Detectable in Healthy Peripheral and Cord Blood. Cytometry Part A 08/2015; 89(3)., DOI:10.1002/cyto.a.22730
10. Lanuti, P., Simeone, P., Rotta, G. et al. A standardized flow cytometry network study for the assessment of circulating endothelial cell physiological ranges. Sci Rep 8, 5823 (2018). https://doi.org/10.1038/s41598-018-24234-0
